# Determinants of serum concentrations of organochlorine compounds in Swedish pregnant women: a cross-sectional study

**DOI:** 10.1186/1476-069X-6-2

**Published:** 2007-02-01

**Authors:** Anders Glynn, Marie Aune, Per Ola Darnerud, Sven Cnattingius, Rickard Bjerselius, Wulf Becker, Sanna Lignell

**Affiliations:** 1 Swedish National Food Administration, P.O. Box 622, SE-751 26 Uppsala, Sweden; 2 Department of Environmental Toxicology, Uppsala University, Norbyvägen 18A, SE-752 36 Uppsala, Sweden; 3 Department of Medical Epidemiology and Biostatistics, Karolinska Institutet, SE-171 77 Stockholm, Sweden

## Abstract

**Background:**

We performed a cross-sectional study of associations between personal characteristics and lipid-adjusted serum concentrations of certain PCB congeners and chlorinated pesticides/metabolites among 323 pregnant primiparous women from Uppsala County (age 18–41 years) sampled 1996–1999.

**Methods:**

Extensive personal interviews and questionnaires about personal characteristics were performed both during and after pregnancy. Concentrations of organochlorine compounds in serum lipids in late pregnancy were analysed by gas chromatography. Associations between personal characteristics and serum levels of organochlorine compounds were analysed by multiple linear regression.

**Results:**

Participation rate was 82% (325 of 395 women). Serum concentrations of PCB congeners IUPAC no. 28, 52, 101, 105 and 167, and *o*, *p'*-DDT and -DDE, *p*, *p'*-DDT and -DDD, oxychlordane, and γ- and α-HCH were in many cases below the limit of quantification (LOQ). No statistical analysis of associations with personal characteristics could be performed for these substances. Concentrations of PCB congeners IUPAC no. 118, 138, 153, 156 and 180, HCB, β-HCH, *trans*-nonachlor and *p*, *p'*-DDE increased with increased age and were highest in women sampled early during the 4 year study period. This shows that older women and women sampled early in the study had experienced the highest life-time exposure levels, probably mainly during childhood and adolescence. The importance of early exposures was supported by lower PCB concentrations and higher β-HCH and *p*, *p'*-DDE concentrations among women born in non-Nordic countries. Moreover, serum concentrations of certain PCBs and pesticide/metabolites were positively associated with consumption of fatty fish during adolescence, and concentrations of CB 156, CB 180 and *p*, *p'*-DDE increased significantly with number of months women had been breast-fed during infancy. Short-term changes in bodily constitution may, however, also influence serum concentrations, as suggested by negative associations between concentrations of organochlorine compounds and BMI before pregnancy and weight change during pregnancy.

**Conclusion:**

Although some of the associations could be caused by unknown personal characteristics confounding the results, our findings suggest that exposures to organochlorine compounds during childhood and adolescence influence the body burdens of the compounds during pregnancy.

## Background

Concentrations of organochlorine compounds, such as polychlorinated biphenyls (PCBs, industrial chemical) and the insecticide DDT and its metabolites, have declined in the environment and foodstuffs in many parts of the world. Background *in utero *exposure to the environmental pollutants may, however, still be a risk factor for neurological, hormonal and immunological effects in infants and children [1-4]. Serum/plasma concentrations of organochlorine compounds are often used in assessment of body burdens of the compounds among pregnant women, and of fetal exposure [5-8]. Studies of concentrations of organochlorine compounds in serum/plasma of pregnant women often report large inter-individual variations in concentrations [9]. The reasons behind this variation are still to a large extent unknown, although several studies have investigated if lifestyle/medical factors can explain at least some of the variation [10-12]. Such determining factors may confound results in epidemiological studies. Moreover, a better understanding of determinants of body burdens of organochlorine compounds during pregnancy will increase possibilities for future actions and recommendations, with the purpose to lower body burdens during pregnancy.

We analysed serum concentrations of 10 PCB congeners and 11 chlorinated pesticides/metabolites among pregnant primiparous women living in Uppsala County, Sweden, 1996–1999. We report determinants of serum concentrations of 5 of these PCB congeners and four of the chlorinated pesticides/metabolites. The concentrations of the other organochlorine compounds analysed were in many cases below the limit of quantification which made it impossible to study associations with personal characteristics of the women. We selected personal characteristics previously reported to be associated with serum/plasma/breast milk concentrations of organochlorine compounds, such as age, year of sampling, BMI, body weight change, country of birth, smoking and alcohol intake [12-16]. Associations with indices of high exposure to organochlorine compounds, due to fish consumption during childhood were also studied, i.e. growing up in a family with a family member involved in commercial fishing or sport-fishing (fisherman/sportfisher family), and growing up along the coast of the contaminated Baltic Sea. Since associations between breast milk exposure during infancy and blood concentrations of organochlorine compounds have been found in adolescents [17], we also studied associations between the number of months the woman had been nursed during infancy. Finally, we analysed associations between serum concentrations of organochlorine compounds and dietary habits the year of pregnancy and the year women attended 7th grade in school (13–14 years of age in Sweden). Influences of dietary habits during the teenage years were studied since concentrations in food were higher in the 1970s-80s than in the mid-late 1990s when the women got pregnant [18-20]. In an attempt to decrease variation in serum concentrations, we only studied women having their first baby. Nursing is a major pathway of excretion of organochlorine compounds [21].

The aim of our study was to find major determinants of body burden of organochlorine compounds during pregnancy, in order to improve the understanding of reasons behind inter-individual variation in body burdens.

## Methods

### Study population

From January 1996 to May 1999, 1037 pregnant women living and seeking prenatal care in Uppsala County were asked to participate as controls in a case-control study of risk factors for early miscarriages [22]. In all, 953 women (92%) accepted this offer. All women were Swedish-speaking, and had completed 6–12 weeks of pregnancy when entering the study. At 32–34 gestational weeks 50 women had been lost from the study due to miscarriage, induced abortions, and due to mothers withdrawing from the study, moving outside Uppsala County or being lost in follow-up [22].

All primiparas recruited between early fall 1996 and spring 1999 were in late pregnancy (week 32–34) asked to participate in the organochlorine compound study (n = 370). In order to increase the number of women living along the coast of the Baltic Sea in the study population, all Swedish-speaking primiparas (n = 25) at the prenatal clinic in Östhammar, that were not participating in the case-control study, were in early pregnancy (week 6–12) asked to participate (between fall of 1997 and spring of 1999).

Of the 395 women asked to participate in the organochlorine compound study, 325 women (82%) agreed to donate a serum sample in late pregnancy (week 32–34) for chemical analysis, 305 women from the case-control study and 20 women from the Östhammar clinic. Two samples were lost before chemical analysis. Age of the participating 323 women ranged from 18 to 41 years (Table 1). Participation did not occur until after informed consent was obtained. The Ethics Committee of the Medical Faculty, Uppsala University, approved the study (dnr 96114).

**Table 1:** Personal characteristics of the participating primiparous pregnant women.

Variable	N	Mean (SD)	%
Age (yr)	323	28 (4)	
Pre-pregnancy BMI (kg/m^2^)	315	23.2 (3.7)	
Weight increase during pregnancy (%/week)	315	0.61 (0.25)	
Breast-fed during infancy (months)	170	4 (3)	
Smoking during pregnancy	321		20
Alcohol during pregnancy	323		17
Childhood in a fishermen/angler family	213		12
Lived on east coast of Sweden ≥5 yr	223		25
Born in a Nordic country	323		95
Years of education	323		
≤11 years			28
12–13 years			21
14–16 years			25
≥16 years			25

SD, standard deviation

### Interviews and questionnaires

In-person interviews, using a standard structured questionnaire, were conducted by certified midwives at 6–12 and 32–34 completed gestational weeks. Data on maternal characteristics included age, weight before pregnancy, years of education, home address, country of birth, and alcohol consumption and smoking during pregnancy. Consumption of low alcohol beer, cider, medium alcohol beer, high alcohol beer, wine, and hard liquor was quantified as number of drinks per week. Women gave information about consumption before pregnancy and during the week before the interview. Regarding smoking habits women were asked to give information about how many cigarettes they had smoked per week starting four weeks before the pregnancy date. The length of the women were measured and women were weighed on both interview occasions. Blood samples for cotinine analysis (indicator of smoking habits) were taken on both occasions.

After agreeing to participate in the organochlorine compound study, mothers answered a self-administered questionnaire including questions about dietary habits (see below), and delivery (weight of the mother at delivery, birth weight of the child, etc). Moreover, the questionnaire included questions about personal characteristics not covered by the interviews, such as where the women had lived during childhood and adolescence, if they had grown up in a fisherman/sportfisher family, and the number of months the women had been nursed as infants.

### Dietary habits

Dietary questions were designed to collect information about consumption of food groups that are major contributors to dietary exposure to organochlorine compounds, i.e. meat and meat products (including poultry), dairy products, eggs and egg products, fish and fish products and vegetable oils. The food frequency questionnaire included 62 food items. Participants were asked about consumption of different types of foods during the year they got pregnant and during the year they attended 7th grade in school. For fish, meat, eggs and some dairy products we used nine predefined frequency categories ranging from "never" to "once or more per day". Questions about milk and cheese consumption were open-ended and we asked about number of glasses of milk, and slices or table spoons of cheese, consumed per day. The questionnaire also included questions about the type of fat commodity used at the table, and the type of fat used for food preparation. We also asked about the amount (no. of table spoons) used for food preparation and thickness of the fat layer on sandwiches (thick, thin, very thin or no fat). For consumption calculations (g of food stuff/d) we used standard portion sizes [23,24], and lipid content of dairy products were obtained from [25].

In questions about fish consumption, single fish species were identified. The questionnaire provided information on whether the fish was caught by the participant (or a friend of the participant) or was bought in a store/market. Information about the origin of fish was also obtained. Concentrations of organochlorine compounds in fish varies considerably, depending on fat content of the fish and place of catch (fresh water fish, Baltic Sea fish, other marine fish) [26]. Consumed fish were therefore divided into three groups: (1) lean fish consisting of cod-like species, flat-fish, canned fish (except herring), pike, pike-perch, perch, burbot and fish products such as fish sticks and fish quenelles; (2) fatty Baltic Sea fish consisting of Baltic herring and wild salmon/trout; (3) other fatty fish consisting of Atlantic herring, mackerel, Pacific salmon, farmed salmon/trout, whitefish and eel.

### Blood sampling and chemical analysis

Blood was sampled in late pregnancy (week 32–34). We analysed the lipid portion of serum samples for chlorinated pesticides/metabolites *p*, *p'*-DDT, *p*, *p'*-DDD, *p*, *p'*-DDE, *o*, *p'*-DDT, *o*, *p'*-DDE, HCB, α-, β- and γ-HCH, *trans*-nonachlor and oxychlordane (Table 2). We chose to analyse PCB congeners with IUPAC nos. 28, 52, 101, 105, 118, 138, 153, 156, 167 and 180. The analytical method used is described in detail by Atuma and Aune [27]. After extraction and clean up of samples, they were analysed on a gas chromatograph with dual capillary columns and electron capture detectors (^63^Ni). The columns were of different polarity to ease identification of analytes, which was based on retention times relative to internal standards. Quantification was performed using multi-level calibration curves.

**Table 2:** Serum concentrations of PCB congeners and chlorinated pesticides/metabolites^a^.

PCB	Concentration (ng/g lipid)		
	Mean	Min-max	95th percentile

CB 28	1	1–423	17
CB 52	1	1–166	3
CB 101	1	1–183	3
CB 105	1	1–24	4
CB 118	11	3–93	27
CB 138	29	6–100	59
CB 153	59	14–179	127
CB 156	4	1–27	10
CB 167	1	1–9	4
CB 180	38	8–139	78
Hexachlorobenzene	23	12–163	38
γ-Hexachlorocyclohexane	1	1–8	1
α-Hexachlorocyclohexane	1	1–7	1
γ-Hexachlorocyclohexane	9	3–60	22
Oxychlordane	3	1–22	6
*trans*-Nonachlor	5	6–23	12
*o*, *p'*-DDT	2	2–2	2
*o*, *p'*-DDE	2	2–2	2
*p*, *p'*-DDT	5	2–124	18
*p*, *p'*-DDD	2	2–19	2
*p*, *p'*-DDE	88	2–622	316

^a^N = 321–323

Limit of quantification was 2–4 ng/g lipid. Reproducibility was demonstrated by 21 replicate determinations using an in-house control serum sample, included among analytical batches. Coefficients of variation (CV) was less than 13% for most of the compounds, except for CB 105 (20%) and CB 28 (22%). CV for gravimetric fat content was 4%. Average recoveries of different PCB congeners in spiked serum samples were 98 ± 12% (mean ± SD) and 94 ± 8% for 0.1 and 0.8 ppb concentrations, respectively. Recoveries for the chlorinated pesticides varied from 78 to 118%. This shows that loss of compounds during the analytical process was negligible. Results reported were not corrected for recovery. When concentrations were below LOQ they were set to 50% of that limit in all statistical analysis.

### Calculations and statistical analysis

Serum concentrations of organochlorine compounds were lipid adjusted and statistical analyses were performed on logarithmically transformed data, since the distribution of data closely followed a log-normal distribution. Level of significance was set to <0.05 in all tests. Concentrations of CB 28, CB 52, CB 101, CB 105, CB 167, α- and γ-HCH, oxychlordane, *p*, *p'*-DDT, *p*, *p'*-DDD, *o*, *p'*-DDT, and *o*, *p'*-DDE were in many cases (>20%) below the LOQ. Although results of the serum analysis is presented for these compounds, statistical analysis of associations between concentrations and personal characteristics was not performed for these compounds.

Multiple regression (MINITAB^® ^For Windows, 12.22) was used to analyse associations between the dependent variable "organochlorine compound concentration" and independent variables suspected to be determinants of organochlorine compound concentrations. In the first step all independent variables were included in the regression model. In the next step a "basic" model was used, including independent variables with few missing observations (among < 10% of the women), and that were significantly associated with concentrations of most of the organochlorine compounds (p < 0.05) in the first regression. The basic model included the independent variables age of the women, sampling year (starting point Jan. 1 1996), pre-pregnancy BMI and weight change during pregnancy. Independent variables with many missing values were then included one at a time in the basic model. This procedure minimized the loss of power in the statistical analysis.

Determinants studied, using the basic regression model described above, were education level, smoking, alcohol consumption, country of birth, growing up in a fisherman/sportfisher family or along the Baltic Sea coast, and breast milk exposure during infancy. Weight change during pregnancy was calculated as % per week, from pre-pregnancy weight to weight at blood sampling (week 32–34). Smoking was categorized into three categories, never smoked, stopped smoking before pregnancy, and smoked during pregnancy. Women with cotinine concentrations >15 ng/ml in early and/or late pregnancy were classified as smokers during pregnancy, even if they reported that they had not smoked during pregnancy [22]. The variable country of birth was divided into two categories, women born in Nordic countries (Sweden, Denmark, Finland, Norway and Iceland) and women born in non-Nordic countries. In regression analysis observations with standardized residuals ≥3 were omitted because of their exaggerated influence on regression results.

Regression analysis of associations between food habits and concentrations of organochlorine compounds only included data on Nordic-born women that had completed the food frequency questionnaire (N = 226). The basic regression model, described above, was used, and associations between organochlorine compound concentrations and consumption of each food group (g/day) were analysed one at a time. Consumption was, with two exceptions, divided into four categories, with an effort to have equal number of individuals in the categories. Consumption of fatty Baltic fish was divided into three categories, including women with no consumption in the first category, and remaining women evenly divided into two other categories. Egg consumption was also divided into three categories, with an effort to have equal number of individuals in each category.

Stepwise regression was used to estimate how much of the variation in organochlorine compound concentrations that was explained by the different determining variables. Adjusted geometric means of organochlorine compound concentrations were calculated using the general linear model (GLM) procedure.

Partial regression coefficients (*b*) for independent variables age, year of sampling, pre-pregnancy BMI, weight change during pregnancy, and breast milk exposure during infancy were used in calculation of %change in organochlorine compound concentration per unit change of determining variables:

%change = (1-exp(*b*))*100.

## Results

### Concentrations of organochlorine compounds

Median serum lipid concentrations were highest for PCB congeners CB 138, CB 153 and CB 180, and for HCB and the DDT metabolite *p*, *p'*-DDE (Table 2). Among PCB congeners, CB 28, CB 52 and CB 101 showed the largest variation (>100-fold), whereas *p*, *p'*-DDT exhibited the largest variation among chlorinated pesticides/metabolites (approx. 60-fold) (Table 2). A few women had high concentrations of CB 28, CB 52 and CB 101 (Table 2).

### Lifestyle/medical factors

Multiple regression analysis showed statistically significant associations between organochlorine compound concentrations and age of the women (all substances, positive), year of sampling (all substances, negative), pre-pregnancy BMI (CB 138, CB 153, CB 156, CB 180, *trans*-nonachlor, negative), and weight change during pregnancy (all substances except β-HCH and *p*, *p'*-DDE, negative) (Table 3). A regression model including these determinants explained 50–70% (R^2^, coefficient of determination) of the variation in organochlorine compound concentrations. Associations were strongest for age (R^2^:27–52%), whereas year of sampling explained 3–11% of the variation in serum concentrations. Pre-pregnancy BMI and weight change during pregnancy showed a weaker association, on average explaining 0.5–4% of the variation in organochlorine compound concentrations (Table 3).

**Table 3:** Percent change in serum concentrations per unit change of determining factors^a^.

Substance	Age (years)	Sampling year	Pre-pregnancy BMI (kg/m^2^)	Weight gain (% per week)	Breast-fed^b ^(months)
	(%change)	(%change)	(%change)	(%change)	(%change)

CB 118	7.9 (7.4, 8.5)*	-16 (-18, -13)*	-0.9 (-0.3, -1.5)	-28 (-34, -22)*	0.6 (-0.3, 1.5)
CB 138	8.0 (7.5, 8.6)*	-13 (-15, -11)*	-1.3 (-1.9, -0.7)*	-16 (-22, -8)*	1.1 (0.3–1.9)
CB 153	8.5 (8.1, 9.0)*	-12 (-14, -11)*	-2.9 (-3.4, -2.4)*	-24 (-30, -18)*	1.3 (0.5, 2.1)
CB 156	13 (12, 13)*	-16 (-19, -14)*	-4.5 (-5.3, -3.8)*	-35 (-42, -27)*	2.3 (1.2, 3.5)*
CB 180	9.0 (8.6, 9.4)*	-12 (-14, -10)*	-3.6 (-4.1, -3.2)*	-32 (-36, -27)*	1.4 (0.7, 2.0)*
HCB	3.6 (3.3, 4.0)*	-11 (-13, -9)*	-0.4 (-1.0, 0.2)	-22 (-26, -18)*	0.3 (-0.3, 0.9)
β-HCH	6.4 (5.9, 6.9)*	-16 (-18, -14)*	0.4 (-0.2, 1.0)	-11 (-19, -3)	1.3 (0.5, 2.1)
TN^c^	7.6 (7.0, 8.3)*	-18 (-20, -15)*	-3.0 (-3.6, -2.4)*	-38 (-44, -32)*	1.0 (0.1, 1.9)
*p*, *p'*-DDE	8.8 (7.8, 9.3)*	-13 (-16, -10)*	0.2 (-0.6, 1,0)	-10 (-21, 2)	3.4 (2.2, 4.7)*

^a^Adjusted means (-SD, +SD) calculated from the partial regression coefficients using a regression model including the independent variables age at sampling, year of sampling, pre-pregnancy BMI and weight gain during pregnancy.

^b^The regression model also included number of months the women were breast-fed during infancy.

^c^*trans*-Nonachlor.

*p > 0.05, N = 170–312.

**Figure 1:**
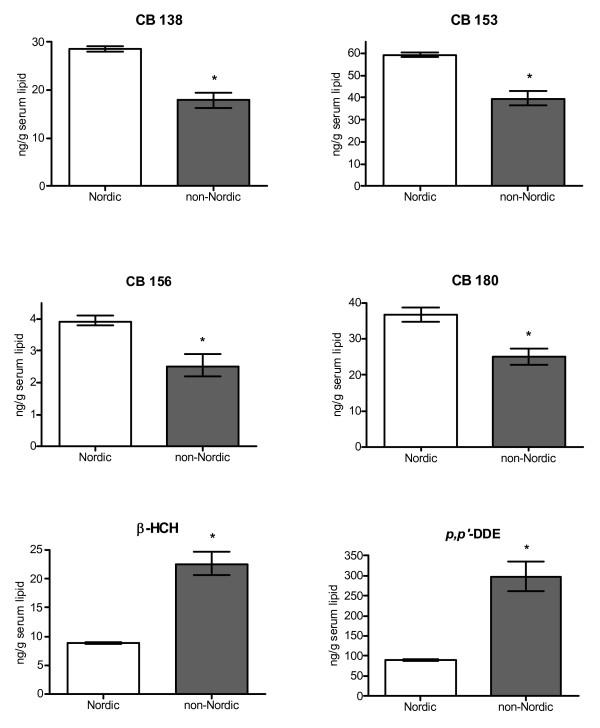
Adjusted geometrical means (± SD) of PCB and chlorinated pesticide/metabolite concentrations in serum lipids (ng/g lipid) among primiparous women born in Nordic (N = 307) or non-Nordic countries (N = 16). Adjusted for age, year of sampling, pre-pregnancy BMI, and weight change during pregnancy. Results shown are for organochlorine compounds with significantly different adjusted means between Nordic (N = 307) and non-Nordic women (N = 16) (p ≤ 0.05).

Regression analysis also showed that women born in countries outside the Nordic region had 1.3–1.6-fold lower adjusted mean concentrations of CB 138, CB 153, CB 156 and CB 180 than women born in the Nordic countries (R^2^: 2–4%) (Figure 1). On the contrary, higher adjusted mean concentrations of β-HCH (2.4-fold) and *p*, *p'*-DDE (3.3-fold) were found among non-Nordic women compared to Nordic women (R^2^: 16–17%) (Figure 1).

A weak (R^2^: 1–3%), but statistically significant, positive association was found between number of months women had been breast-fed during their infancy and serum concentrations of CB 156, CB 180 and *p*, *p'*-DDE (Table 3). Other potential determining variables (education, smoking, living in a fishermen/sportfisher family, living on the east coast for more than 5 years, and alcohol consumption during pregnancy) showed no statistically significant associations with serum organochlorine compound concentrations (results not shown), except for alcohol consumption and concentrations of *trans*-nonachlor, and education level and HCB. In this case the adjusted mean concentration was 14.0% (SD:7.4–20.9%, p ≤ 0.05) higher among women consuming alcohol during pregnancy than among those not consuming alcohol. Moreover, women with the highest education level had a 13.0 % (SD: 8.5–17.6%, p ≤ 0.05) higher adjusted geometric mean of HCB than women with lowest level of education.

### Dietary habits

Consumption of lean fish dominated the fish consumption, and more than 50% of the women did not eat fatty fish from the Baltic Sea. The women reported higher food consumption rates in 7th grade, except for consumption of other fatty fish (Table 4).

**Table 4:** Self-reported food consumption among pregnant primiparous women (mean (SD)).

Food products	Pregnancy year (g/day)	Adolescence (g/day)^a^	Difference (g/day)^b^
Meat, meat products	103 (47)	113 (49)	12 (49)*
Milk fat	26 (14)	31 (20)	6 (17)*
Vegetable fat	13 (10)		
Eggs	12 (9)	14 (9)	3 (9)*
Fish total	24 (20)	32 (24)	8 (21)*
Lean fish	18 (15)	23 (18)	6 (17)*
Fatty Baltic fish	1 (3)	3 (5)	2 (5)*
Other fatty fish	5 (6)	5 (6)	-0.3 (6)

^a^The year women attended 7th grade in school. In Sweden 13–14 years old. Data for vegetable fat missing since no questions about vegetable fat was asked for this time period.

^b^Difference between consumption during adolescence and consumption during the year they became pregnant.

*Wilcoxon Signed Rank Test, p ≤ 0.05, N = 222–226.

**Figure 2:**
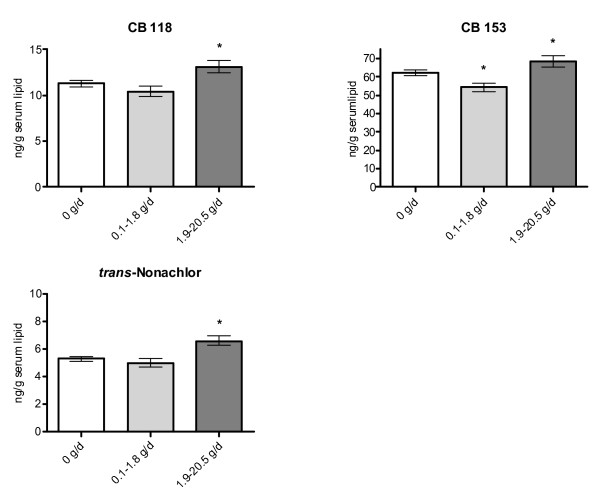
Adjusted geometrical means (± SD) of PCB and chlorinated pesticide/metabolite concentrations in serum lipids (ng/g lipid) among primiparous women with different consumption rates (g/day) of herring and wild salmon/trout from the Baltic Sea (fatty Baltic fish) the year they became pregnant. Adjusted for age, year of sampling, pre-pregnancy BMI, and weight change during pregnancy. Statistical analysis performed only on women born in Nordic countries. Results shown are for organochlorine compounds with significantly different adjusted means between women with the lowest and highest consumption levels (p ≤ 0.005, N = 226).

Among food groups studied, total fish consumption, fatty Baltic fish consumption, and other fatty fish consumption during the year of pregnancy were positively associated with CB 118 serum concentrations (Table 5, Figure 2). Consumption of fatty Baltic fish was positively associated with CB 153 concentrations, and *trans*-nonachlor concentrations were positively associated with total fish consumption. Less than 3% of the variation in serum concentrations was explained by the variation in fish consumption. No other positive or negative associations between food consumption during the year of pregnancy and serum concentrations were found (results not shown).

**Table 5:** Serum organochlorine compound concentrations in pregnant women and consumption of fish the year of pregnacy^a^.

Consumption (g/day)	N	CB 118 (ng/g lipid)	*trans*-Nonachlor (ng/g lipid)
Fish total^b^			
0–8.0	56	10.6 (10.1–11.1)	5.2 (4.9–5.5)
8.1–21.4	57	11.3 (10.8–11.9)	5.5 (5.3–5.8)
21.5–32.2	56	11.6 (11.0–12.1)	5.2 (4.9–5.4)
32-3-106.7	57	12.2 (11.7–12.8)*	6.0 (5.7–6.3)*
Other fatty fish^c^			
0–0.9	58	10.2 (9.7–10.6)	
1.0–2.4	55	11.8 (11.2–12.4)*	
2.5–6.9	53	11.8 (11.2–12.3)*	
7.0–30.0	60	12.2 (11.6–12.7)*	

^a^Geometric mean (± standard deviation) adjusted for age, year of sampling, pre-pregnancy BMI, and weight change during pregnancy. Statistical analysis performed only on women born in Nordic countries. Results are shown only for organochlorine compounds that had adjusted means that were significantly different between groups with lowest and highest consumption.

^b^Including lean fish, fatty fish from the Baltic Sea and other fatty fish.

^c^Fatty fish originating from other areas than the Baltic Sea.

*Significantly different from the group with lowest consumption, p ≤ 0.05.

**Figure 3:**
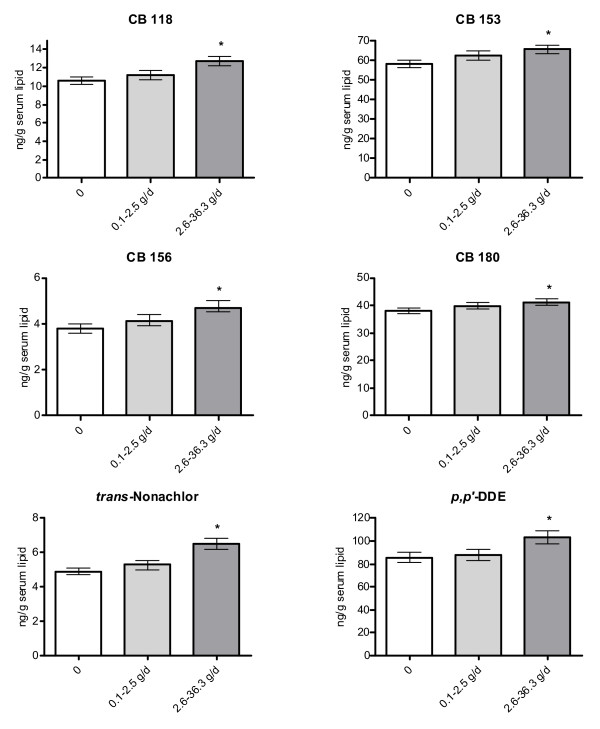
Adjusted geometrical means (± SD) of PCB and chlorinated pesticide/metabolite concentrations in serum lipids (ng/g lipid) among primiparous women with different consumption rates (g/day) of herring and wild salmon/trout from the Baltic Sea (fatty Baltic fish) the year they attended 7th grade in school. Adjusted for age, year of sampling, pre-pregnancy BMI, and weight change during pregnancy. Statistical analysis performed only on women born in Nordic countries. Results shown are for organochlorine compounds with significantly different adjusted means between women with the lowest and highest consumption levels (p ≤ 0.05, N = 226).

**Figure 4:**
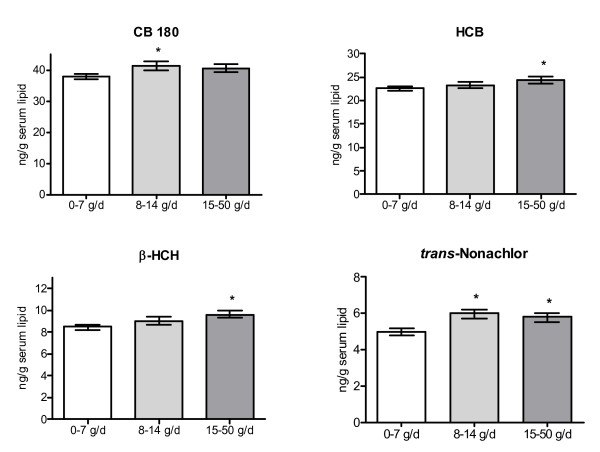
Adjusted geometrical means (± SD) of PCB and chlorinated pesticide/metabolite concentrations in serum lipids (ng/g lipid) among primiparous women with different consumption rates (g/day) of eggs the year they attended 7th grade in school. Adjusted for age, year of sampling, pre-pregnancy BMI, and weight change during pregnancy. Statistical analysis performed only on women born in Nordic countries. Results shown are for organochlorine compounds with significantly different adjusted means between women with the lowest and highest consumption levels (except for CB 180).

Similar results were found for the 7th grade consumption, although results for fish consumption during adolescence was statistically significant for a few more organochlorine compounds than was the case for consumption during the year women became pregnant (Table 6, Figure 3). Moreover, a significantly higher adjusted mean concentration of HCB, β-HCH and *trans*-nonachlor was found among women in the highest consumption of eggs and egg products compared to women in the lowest consumption group (Figure 4). No significant associations were found for other food groups (results not shown).

**Table 6:** Serum organochlorine compound concentrations in pregnant women and consumption of fish in 7th grade^a^.

Consumption (g/day)	N	CB 118 (ng/g lipid)	CB 156 (ng/g lipid)	HCB (ng/g lipid)	*trans*-Nonachlor (ng/g lipid)
Fish total^b^					
0–16.6	55	10.2 (9.7–10.7)	3.7 (3.5–4.0)	19.8 (20.3–21.6)	5.1 (4.8–5.3)
16.7–26.2	57	11.4 (10.8–11.9)	4.1 (3.8–4.3)	24.5 (23.8–25.3)*	5.3 (5.0–5.6)
26.3–40.1	56	11.6 (11.1–12.1)	4.4 (4.2–4.7)*	23.7 (23.0–24.4)*	5.5 (5.2–5.8)
40.2–297.1	57	12.3 (11.8–12.9)*	4.4 (4.2–4.7)*	24.0 (23.3–24.8)*	6.0 (5.7–6.3)*
Other fatty fish^c^					
0–0.9	55	10.6 (10.1–11.1)			
1.0–2.4	56	11.2 (10.8–11.8)			
2.5–6.9	56	11.5 (10.9–12.0)			
7.0–30.0	57	12.2 (11.6–12.8)*			

^a^Geometric mean (± SD) adjusted for age, year of sampling, pre-pregnancy BMI, and weight change during pregnancy. Statistical analysis performed only on women born in Nordic countries. Results are shown only for organochlorine compounds that had adjusted means that were significantly different between groups with the lowest and highest consumption.

^b^Including lean fish, fatty fish from the Baltic Sea and other fatty fish.

^c^Fatty fish originating from other areas than the Baltic Sea.

*Significantly different from the group with lowest consumption, p ≤ 0.05.

## Discussion

Our results show that several of the personal characteristics of the pregnant primiparous women were similarly associated with lipid-adjusted serum concentrations of many of the studied PCB congeners and chlorinated pesticides/metabolites. This shows that the exposure patterns, body distribution, metabolism, and excretion of the studied organochlorine compounds are similar. However, associations between serum concentrations and pre-pregnancy BMI, weight change during pregnancy, and country of birth varied between compounds. This indicate that there are differences in sources of exposure and toxicokinetics between some of the compounds that should be accounted for in epidemiological studies.

Regression models including the personal characteristics studied by us left 30–60% of the variation in serum organochlorine compound concentrations unexplained. The capacity of a regression model to explain the variation in serum organochlorine compound concentrations is both dependent on number of variables in the model and number of observations in the analysis. Most determining variables, studied by us, were not the direct cause of the variation in serum concentrations, instead they are proxy variables for differences in exposures, toxicokinetics, etc. It can therefore be difficult to draw conclusions about the reasons behind the associations.

Moreover, the use of interviews and questionnaires as a method of information collection adds uncertainty to some of the determinants. For instance, it must have been difficult for the women to give correct answers regarding weight before pregnancy, smoking habits, alcohol consumption, months of being breast-fed as an infant etc. Bias in the answers about these types of personal characteristics can lead to difficulties in detection of real associations in regression analysis, or to detection of false associations. For instance, answers about smoking habits during pregnancy can be unreliable, since many women may find it embarrassing to admit that they have been smoking during pregnancy. In this case we measured cotinine in blood as a marker of smoking during pregnancy, which improved the reliability of the smoking variable.

In the regression analysis we excluded outliers that had a standardized residual ≥3. In cases of personal characteristics showing a strong association with organochlorine compound concentrations, such as age and year of sampling, the regression results did not change markedly after the outlier had been removed. In some cases, when associations were weak, removal of an outlier had a much larger influence on associations. This further points to the uncertainty of some of the weaker associations. The individuals that were classified as outliers for some of the organochlorine compounds usually had either very low or high serum concentrations. These individuals could differ from the majority of the study participants for instance regarding sources of exposure or metabolic capacity.

The majority of women had very low concentrations of the PCB congeners CB 28, CB 52, CB 101, CB 105 and CB 167, and α- and γ-HCH, oxychlordane, *o*, *p'*-DDT, *o*, *p'*-DDE, *p*, *p'*-DDT and *p*, *p'*-DDD. This indicates that the women had not recently been exposed to the technical mixtures of these different compound groups. A few women, however, had high concentrations of some of the compounds. For instance, serum concentrations of PCB congeners CB 28, CB 52 and CB 101 were ≥20 ng/g lipid in 17 women, and 2 women had concentrations over 100 ng/g lipid. The quantification of the samples with the highest concentrations of CB 28, CB 52 and CB 101 were confirmed by gas chromatography/electron capture negative ion mass spectroscopy. Other serum samples (and quality control samples) in the same analytical batch as the samples with very high concentrations had low concentrations. This excludes the possibility of systematic contamination of certain analytical batches.

We hypothesize that exposure to technical PCB mixtures in building materials in home or working environments could contribute to the high concentrations observed [28-31]. In a study of 21 residents in houses built with building sealants containing high levels of PCBs, the median blood concentration of CB 28 was reported to be 89 ng/g lipid, with a range of 6 to 360 ng/g lipid [29] (Personal communication: Annika Hanberg, Institute of Environmental Medicine, Karolinska Institutet, Stockholm, Sweden). The highest concentrations reported for the PCB-house residents were in the same range as the highest concentrations found by us. In Sweden during the period 1956–1972, 70–190 tons of PCBs were used in sealants between concrete blocks and around doors and windows on the outside of buildings [29]. We did not have access to information about year of construction for all the houses the participating women lived in at the time of sampling. Among women living in the City of Uppsala, 11 women had concentrations >20 ng/g lipid of at least one of the three congeners CB 28, CB 52 and CB 101. Of these women, 5 lived in houses built before the "PCB period", 4 lived in houses built between 1956–1972, and 2 lived in houses built after 1972. Thus, no strong association was evident between high exposure and residence in housing built during the PCB period. We have no information about the presence of PCB in building materials in houses women were living in at the time of blood sampling. Furthermore, information is lacking about PCB status in houses that women had lived in before and in working environments.

As evident from other studies [11,14,32], age at sampling was a strong determinant of serum organochlorine compound concentrations, after adjustment for other important determinants. For instance, a 6-fold difference in adjusted mean concentration of CB 153 was found between 18-year-old and 41-year-old women. Age-related bioaccumulation of the persistent and lipophilic compounds probably contributed to this age dependency of serum concentrations. A birth cohort effect probably also contributed, since environmental and food levels of organochlorine compounds decreased in Sweden during the 1960s-1990s [18,19,33]. Women born in the 1960s and early 1970s thus experienced higher levels of exposure during childhood and adolescence than women born in the late 1970s to early 1980s.

The observed decline in concentrations of organochlorine compound between 1996 and 1999 was probably mainly caused by the birth-cohort effect. The rate of decline is uncertain due to the short study period. Slower decline rates have been reported in a breast milk trend study in the Stockholm area [16]. Lack of age adjustment of results in the Stockholm study could be one reason for the slower declines. Mean age among participating Stockholm women increased from 27–28 years to 30–31 years from early 1970s to late 1990s [16]. Strong positive associations between age and organochlorine compound concentrations in humans should be accounted for in time trend studies.

Lower adjusted mean concentrations of PCBs and higher mean concentrations of β-HCH and *p*, *p'*-DDE among women born in non-Nordic countries are further evidence of how important exposures during childhood and adolescence are for body burdens of certain organochlorine compounds during pregnancy. We only had complete information about time of residence in Sweden for 8 of the 16 non-Nordic women. Seven of those had lived in Sweden for more than 5 years. The women were born in southern Africa, in the Middle East, in the Balkan region, and in southern Europe, regions where use of insecticides, such as DDT and technical HCH mixtures, has been higher than in the Nordic countries [34-36]. Earlier studies from the USA and Germany have also found elevated concentrations of β-HCH and *p*, *p'*-DDE among individuals from countries with extensive use of DDT and HCH products [11,37]. Moreover, low body burdens of PCBs have previously been found in persons from less industrialized countries [15,37].

Surprisingly, serum concentrations of CB 156, CB 180 and *p*, *p'*-DDE were positively associated with number of months the women were breast-fed early in life. It may have been difficult for the participating mothers to get correct information about breast milk exposure during their infancy. Moreover, many women did not answer the breast milk exposure question (153 women out of 323). Nevertheless, our results suggest that high exposures early in life can be traced in the body several decades later. Other studies have shown that breast milk exposure early in life is positively associated with serum concentrations of PCBs, *p*, *p'*-DDE, HCB and β-HCH in children and adolescents [17,38-40].

Negative associations between weight increase during pregnancy and serum concentrations of PCB, HCB and *trans*-nonachlor could be caused by a "dilution" effect of weight gain during pregnancy. Levels of organochlorine compounds in lipids ingested during pregnancy were most probably lower than levels in body lipids at the start of pregnancy. Negative associations between pre-pregnancy BMI and serum PCB and *trans*-nonachlor concentrations could also be due to a dilution effect. Although not studied by us, rapid weight gain before pregnancy could be more common among women with high pre-pregnancy BMI than among those with low BMI. Thus, women with high BMI may have started their pregnancy with more "diluted" serum concentrations. There are, however, many uncertainties regarding the influence of body composition on blood concentrations of organochlorine compounds. Diverging results have previously been found for the associations between pre-pregnancy BMI and blood concentrations of organochlorine compounds during pregnancy [10,11,14].

We found no statistically significant associations between organochlorine compound concentrations in serum and the potential determinants smoking, alcohol consumption and education level except in a few cases (HCB and education, *trans*-nonachlor and alcohol consumption). Moreover, we found no support for our hypothesis that women growing up in fishermen/sportfisher families, or along the Baltic Sea coast for at least five years, would have higher adjusted mean serum concentrations than other women, due to higher exposures to organochlorine compounds from fish.

Fish consumption is a major source of dietary exposure to organochlorine compounds in Sweden [41-43]. Our results confirmed the hypothesis that women reporting high fish consumption, especially contaminated fatty fish from the Baltic Sea, would have higher serum concentrations than women with low consumption. Associations between consumption of fatty Baltic fish during adolescence and body burden of organochlorine compounds were more consistent than associations between body burdens and consumption during the year women became pregnant. Although it can not be excluded that these associations could be due to confounding factors not studied by us, the more consistent associations for consumption during adolescence is supported by higher organochlorine compound concentrations in fish during 1960s to 1980s than in the late 1990s when the women got pregnant [44]. Moreover, the women reported a significantly higher consumption of fatty Baltic fish in 7th grade than during the year of pregnancy. Several other studies on pregnant women have shown that fish consumption during the time period around pregnancy is positively associated with organochlorine compound concentrations in serum/plasma [10,11,45,46].

Median self-reported consumption of meat and fish did not differ between the participating women and 25–34 year old women participating in the national food consumption survey Riksmaten 1997–98 [47]. This suggests that the food consumption reported by the women was representative for the general Swedish population.

Women reporting a high consumption of eggs and egg products during adolescence had higher adjusted mean concentrations of HCB, β-HCH and *trans*-nonachlor than women reporting the lowest level of consumption. Recent intake calculations show that egg consumption is not a major source of intake of organochlorine compounds in the Swedish population [24], and results from the food control in the 1970s did not indicate substantially higher concentrations in eggs than in meat and dairy products [20]. The results could be significant by chance or be due to unknown factors correlated to egg consumption.

## Conclusion

Associations between serum organochlorine compound concentrations and age of the Uppsala women, sampling year and birth place suggest that long-term cumulative exposures have a major impact on body burdens of organochlorine compounds during pregnancy. Furthermore, high exposures early in life (breast milk exposure and fish consumption) may still influence serum concentrations during pregnancy decades later. Several of the determining factors studied by us may be important confounders in epidemiological studies of associations between disease and exposure to organochlorine compounds, and should be accounted for in such studies.

## Abbreviations

BMI, body mass index; CB, chlorinated biphenyl; DDD, dichlorodiphenyldichloroethane; DDE, dichlorodiphenylchloroethane; DDT, dichlorodiphenyltrichloroethane; HCB, hexachlorobenzene; HCH, hexachlorocyclohexane; IUPAC, International Union of Pure and Applied Chemistry; NFA, National Food Administration; PCB, polychlorinated biphenyl; SD, standard deviation.

## Competing interests

The author(s) declare that they have no competing interests.

## Authors' contributions

AG participated in the planning of the study, was responsible for data collection, did the data analysis and wrote the first draft of the manuscript. MA was involved in the planning of the study and was responsible for organochlorine compound analysis. POD participated in the planning of the study and in data collection. SC took part in the planning of the study and helped with data collection. RB was involved in data collection and analysis. WB helped with planning of the study and data analysis. SL helped with data analysis. All authors participated in the preparation of the final manuscript and approved the submission.
